# The Claims of Hospitals

**Published:** 1888-09-01

**Authors:** R. Brudenell Carter

**Affiliations:** Ophthalmic Surgeon to St. George's Hospital and the National Hospital for the Paralysed and Epileptic; being a revised edition of the Mansion House Address delivered two years ago.


### THE CLAIMS OF HOSPITALS.

By R. BRUDENELL CARTER, F.R.C.S., Ophthalmic Surgeon to St. George's Hospital and the National Hospital for the Paralysed and Epileptic; being a revised edition of the Mansion House Address delivered two years ago.

As President of the Medical Society of London, at once the oldest and the most comprehensive of the various voluntary associations which have been established for the cultivation of medical science, I may claim to express with some authority the convictions entertained by medical men with regard to the importance to the community of the efficient maintenance of hospitals. I am very anxious to dispel an erroneous belief, which, although erroneous, is very generally entertained, to the effect that hospitals should be looked upon as places supported by the comparatively rich, from motives of benevolence towards the comparatively poor; and that the latter, as represented by the actual patients, are the persons whom hospitals are intended to benefit, and to whom, indeed, their benefits are almost confined. Such a belief owes its origin in no small measure to the style of the so-called "appeals" to the charitable and benevolent, with which we are only too familiar; and which can hardly fail to keep alive the impression that the rich have no concern with hospitals except in their position of benefactors to them. The very reverse of this proposition would be far nearer the truth; for hospitals are now the sole means of medical education; and, but for them, there would be no doctors at all. There would be no advancement of medical science, for there would be no medical science to advance. The professors of the art of healing, if such there were, would be on the same plane with those who were satirised by Molière, and the pressure of redundant population would be tempered by the periodical recurrences of pestilence.

It is, I hope, superfluous for me to dwell upon what the medical profession, the outcome and offspring of hospitals, has done, say only in the last fifty years, to promote the general welfare of the community. Lord Macaulay, in a well-known passage of his comparison of the England of the Stuarts with that of his own day, says: "Every bricklayer who falls from a scaffold, every sweeper of a crossing who is run over by a carriage, may now have his wounds dressed and his limbs set with a skill such as, a hundred and sixty years ago, all the wealth of a great lord like Ormond, or of a merchant prince like Clayton, could not have purchased. Some frightful diseases have been extirpated by science; and some have been banished by police. The term of human life has been lengthened over the whole kingdom, and especially in the towns. The year 1685 was not accounted sickly; yet in the year 1685 more than one in twenty-three of the inhabitants of the capital died. At present only one inhabitant of the capital in forty dies annually. The difference in salubrity between the London of the nineteenth century and the London of the seventeenth century is very far greater than the difference between London in an ordinary year and London in a year of cholera." Forty years have passed away since the passage which I have quoted was written; and the mortality among Londoners, in 1885, was reduced to one in every fifty-one of the inhabitants. This reduction has been partly due to the diffusion of sanitary knowledge, partly to the better treatment of occurring disease; and in both respects it has been the direct outcome of hospitals. The great discovery of Dr. Budd, of the conveyance of the poison of typhoid fever by water, has shown us how to arrest its ravages. The cholera, from which Lord Macaulay took one of his most telling illustrations, has been robbed, for us, of its terrors; although the experience of a neighbouring country shows that the disease itself has lost none of its virulence. Among the diseases which affect individuals rather than communities, many have been brought within the reach of prevention, and many within the reach of cure. Dr. Quain, in an admirable address which he delivered to the students at Netley, sketched for them the histories of the Walcheren and Crimean campaigns, and drew a contrast between these and the recent campaigns in Egypt. In Walcheren, out of a garrison of 18,000 men, more than half died from disease within three months, so that the place was evacuated. In the Crimea the total mortality among the English troops was 18,058, exclusive of those killed in action. Of this number 16,297 deaths were the result of disease alone, and only 1,761 of wounds or injuries; and it is recorded that in the month of January, 1855, nearly ten per cent. of the troops succumbed to disease. In 1882, out of 13,000 men serving in Egypt, nearly half of whom passed through hospital, the total of deaths, including 93 killed in action, was only 172. In the Suakim force in 1885, with an approximate strength of ten thousand men, there were only 17 deaths out of 2,047 cases admitted into hospital; and in the first Suakim expedition, out of 4,000 men, with 314 admissions to hospital, there were no deaths at all. In earlier Egyptian warfare ophthalmia has been the great scourge of armies, and the French, in 1804, are said to have sent home 1,000 men absolutely deprived of sight; while in our own recent campaigns in the country not a single man was blinded, although nearly 1,500 cases of inflammatory affections of the eyes were admitted into hospital.

While such have been the practical results of the teachings given in hospitals, when regarded from the most comprehensive point of view, I may be permitted to point out that the value of this teaching is not less conspicuous if we look at its effects upon the narrower circle afforded by every medical practitioner and his patients. If a labouring man breaks his leg, he is taken into one of the great London hospitals, and remains there for some weeks, tenderly nursed and cared for. At length he is discharged cured, and able shortly to resume his occupation. His earnings continue as before; and so a good work is done, the effects of which end, so far, with the benefit conferred upon the man, and upon his wife and family. But consider how greatly this good work is extended, and how much larger are its proportions, if the case affords the means of teaching, to each of fifty or of a hundred students, something about the way in which such injuries should be treated; and thus enables each one of them to go forth into the world, and to encounter similar injuries in his turn, better prepared to deal with them than would otherwise have been the case. Surely we are justified in regarding the teaching afforded by a hospital as the most important part of its work, and the cure of the actual patients admitted as something which is secondary in importance, as it is certainly secondary in magnitude. Nor is it only the general or family practitioner, and the patients who are compelled to rely upon his skill, who are the gainers. The teaching power is exercised over a much wider and more important area, in the influence which it has in furnishing the experience and skill which render the possessors of them the trusted advisers not only of the poor, but also of the rich. Without efficient hospitals as schools for students, we should have no capable general practitioners; and without them as schools for physicians and surgeons, we should have no men capable of performing difficult and delicate operations, or of investigating the most obscure conditions of disease.

The circumstances which enable hospitals to become the centres of medical progress, and the great promoters of the growth of medical science, must on no account be misunderstood or misinterpreted. These circumstances are, mainly, the facilities which they afford for observation, whether of the natural history and events of disease, or of the action of remedies. Suppose, for example, that a physician wishes a certain observation, say of pulse, or of temperature, or of any other bodily phenomenon, to be taken with minute accuracy every half hour. In private practice, this would be almost impossible of accomplishment; in a hospital, it at once becomes the duty of one of the clinical assistants. Suppose that the physician wishes the same observation to be made in the case of a large number of patients suffering from the same disease, and living within the some environment, but in other respects differing from one another. The hospital affords him at once the patients, the similarity of environment, and the skilled observers. There can perhaps be no better illustration of my general principle than that which is furnished by the question of temperature. When I was a medical student, and indeed for some years afterwards, it was a received doctrine that the internal temperature of the human body did not vary, and that heat of skin was only skin-deep. I remember, as if it were yesterday, the time when this doctrine was overthrown, and the still later time when the thermometer first became one of the doctor's bedside appliances. It is now an instrument absolutely indispensable to every medical practitioner, one which constantly gives the first warning of impending danger, and the first sign of commencing improvement. That we know what we do about it, how to interpret its indications in different diseases, and in patients at different periods of life, is solely due to the opportunities which hospitals have afforded, and to the indefatigable industry with which these opportunities have been employed. I might give many similar examples, but the principle underlying them would always be the same.

On various other occasions, when I have contended that the greatest use of hospitals is to promote the advancement of medical science, and to afford us improved methods of recognising and of treating disease, I have been met by the well-meaning objection, "Oh, but it cannot be right to try experiments on patients;" and I think it is worth while to look at this phrase somewhat more closely. Much turns, of course, on what we mean by the word experiment: for, in one sense, it is an experiment to do anything which in any degree departs from precedent. But I may say, first, that what I may call the sacredness of the patient is a feeling which underlies the whole course of medical training and practice in this country, far more, I think, than in any other; and that no medical practitioner, certainly none whose previous career entitles him to be entrusted with the responsibilities of a hospital appointment, would ever either do or sanction the doing, to a patient, of anything to which he would not himself submit in like circumstances, or which was not, in his judgment, calculated to be beneficial. Experiment, in this sense of the word, is merely the application of previously acquired knowledge one step further; and hospitals allow such further applications to be made under the most favourable conditions. For instance, when the thermometer had been applied for a time, it was found that certain forms of disease, which tended very rapidly towards a fatal issue, were associated with a great and sudden elevation of temperature. To check and control this elevation of temperature by the careful use of cold baths was, in a sense, an experiment; but it was an experiment which sprang naturally from observation, and which was so plainly beneficial that it at once became a recognised resource in practice, and one which has preserved numbers of valuable lives. It would have been a proper and justifiable experiment anywhere, in a private house as in a hospital, but the opportunities afforded by hospitals rendered it easy to determine the conditions most suitable for its employment, and the manner in which it could be most effectually applied.

Let us suppose that the use of the cold bath in very high temperature had occurred, as a happy thought, or as an inference from previous knowledge, to someone who was not a hospital physician, and had by him been successfully applied. He would no doubt mention it to his professional friends, but it would not be known beyond his immediate circle, and perhaps a twelvemonth might elapse before he would be called to another case which afforded him an opportunity of again employing and testing the method. Anything of this sort which is done in a hospital is at once noticed in the medical journals, is circulated wherever the English tongue is spoken, and becomes part of the knowledge of hundreds of men who are perfectly capable of using it in case of need, but to whom it might never have occurred as an original suggestion.

*(To be continued.)*

